# Multi-Response Optimization in High-Speed Machining of Ti-6Al-4V Using TOPSIS-Fuzzy Integrated Approach

**DOI:** 10.3390/ma13051104

**Published:** 2020-03-02

**Authors:** Adel T. Abbas, Neeraj Sharma, Saqib Anwar, Monis Luqman, Italo Tomaz, Hussien Hegab

**Affiliations:** 1 Mechanical Engineering Department, Engineering College, King Saud University, Riyadh 11421, P.O. Box 800, Saudi Arabia; monisluqman9@gmail.com; 2 Department of Mechanical Engineering, Maharishi Markandeshwar (Deemed to be University), Mullana, Ambala, Haryana 133207, India; neeraj.sharma@live.com; 3 Industrial Engineering Department, Engineering College, King Saud University, Riyadh 11421, P.O. Box 800, Saudi Arabia; sanwar@ksu.edu.sa; 4 Laboratory of Materials Testing (LEMat), Fluminense Federal Institute, Cabo Frio–Buzios, s/n, Cabo Frio RJ 28909-971, Brazil; italo.tomaz@iff.edu.br; 5 Mechanical Design and Production Engineering Department, Cairo University, Giza 12613, Egypt; hussien.hegab@uoit.ca

**Keywords:** machining, Ti-6Al-4V, optimization

## Abstract

Titanium alloys are widely used in various applications including biomedicine, aerospace, marine, energy, and chemical industries because of their superior characteristics such as high hot strength and hardness, low density, and superior fracture toughness and corrosion resistance. However, there are different challenges when machining titanium alloys because of the high heat generated during cutting processes which adversely affects the product quality and process performance in general. Thus, optimization of the machining conditions while machining such alloys is necessary. In this work, an experimental investigation into the influence of different cutting parameters (i.e., depth of cut, cutting length, feed rate, and cutting speed) on surface roughness (Rz), flank wear (VB), power consumption as well as the material removal rate (MRR) during high-speed turning of Ti-6Al-4V alloy is presented and discussed. In addition, a backpropagation neural network (BPNN) along with the technique for order of preference by similarity to ideal solution (TOPSIS)-fuzzy integrated approach was employed to model and optimize the overall cutting performance. It should be stated that the predicted values for all machining outputs demonstrated excellent agreement with the experimental values at the selected optimal solution. In addition, the selected optimal solution did not provide the best performance for each measured output, but it achieved a balance among all studied responses.

## 1. Introduction 

Titanium alloys are widely used in various applications including aerospace, marine, biomedicine, energy, and chemical industries. When compared to other materials, titanium alloys show low density, high hot strength and hardness, and superior corrosion resistance and fracture toughness. Due to the fact of its well-known combination of mechanical and thermal properties, the industrial interest in the use of this type of alloy recently generated a dramatic increase in demand [1,2]. High hot strength and hardness promote premature tool wear and failure. In addition, high chemical reactivity and low thermal conductivity accelerate the tool wear and premature failure [3,4]. Considering that the majority of titanium parts are manufactured by machining processes and have very restricted tolerance and high-quality surface finishing requirements, different industries need to overcome the problems associated with titanium alloy machining. Liang et al. [5] conducted an extensive review discussing the effect of tool wear on surface integrity when machining nickel and titanium alloys and discussed how fast the tool wear could deteriorate the machined surface. They showed evidence that the reduction of contact regions and clearance angle increments due to the tool wear directly changed the heat distribution and stressed state during the cutting process. The additional thermo-mechanical loads generated by the altered heat and stress distribution can affect the mechanical properties, microstructural states, and surface topography. It should be stated that these modifications of machined surface and subsurface areas affect the functional characteristics and lifespan of manufacturing components. Therefore, a machinability assessment should be considered in the development of the manufacturing processes to ensure the desired surface integrity of the machined workpieces as mentioned in the open literature [6,7]. In addition, the selection of appropriate machining parameters is necessary to increase the life of the cutting tool and control the machining costs [8].

Many studies have been conducted in attempts to enhance titanium alloys’ machinability or to identify optimum machining parameters. Wang et al. [9] investigated high-speed milling on titanium-6aluminum-4vanadium alloy using a binderless cubic boron nitride (CBN) cutting tool and observed that an increase in the depth of the cut and feed rate induced higher resultant cutting forces. Although, when the cutting speed was increased, the resultant cutting forces decreased. Kechagias et al. [10] used full factorial and Taguchi design for machinability prediction in the turning of titanium alloys. They also showed that resultant cutting forces increased with an increase in the feed rate and depth of cut and concluded that the design of the experimental technique could effectively be used to evaluate the machinability of such materials. However, these studies focused only on analyzing the influence of each cutting parameter and did not cover surface integrity or machining costs. To optimize the machining processes performance, many multi-objective techniques (e.g., genetic algorithm (GA), particle swarm optimization (PSO), harmony search (HS), artificial bee colony (ABC), ant colony optimization (ACO), fuzzy logic system (FLS)) have been successfully used in the open literature [11,12,13]. Thus, offering an optimized scenario that can cover different machining outputs and aspects is necessary, especially when machining titanium alloy or any other difficult-to-cut material. 

The present study is an investigation into the influence of different machining parameters (i.e., cutting speed (v), feed rate (f), depth of cut (d), and cutting length (l)) on surface roughness (Rz), flank wear (VB), power consumption as well as material removal rate (MRR) when using high-speed turning Ti-6Al-4V alloy. A full factorial experimental design (L48OA) was used to carry out experiments, and then all input and output data were optimized by applying a TOPSIS-fuzzy integrated approach. Such a multi-objective optimization technique is powerful and can offer a reliable solution and a balance among all included outputs. It also helps to provide different solutions that can offer enough flexibility for the decision maker to select the most appropriate cutting conditions based on the desired objectives. 

## 2. Materials and Methods

The material used in this paper was “titanium-6aluminum-4vanadium”. It is used for a wide range of applications such as in marine, power generation, aerospace, and offshore industries. Ti-6Al-4V is the most popular of the titanium alloys. Table 1 shows the chemical composition, while Table 2 shows its mechanical properties.

**Figure 1:**
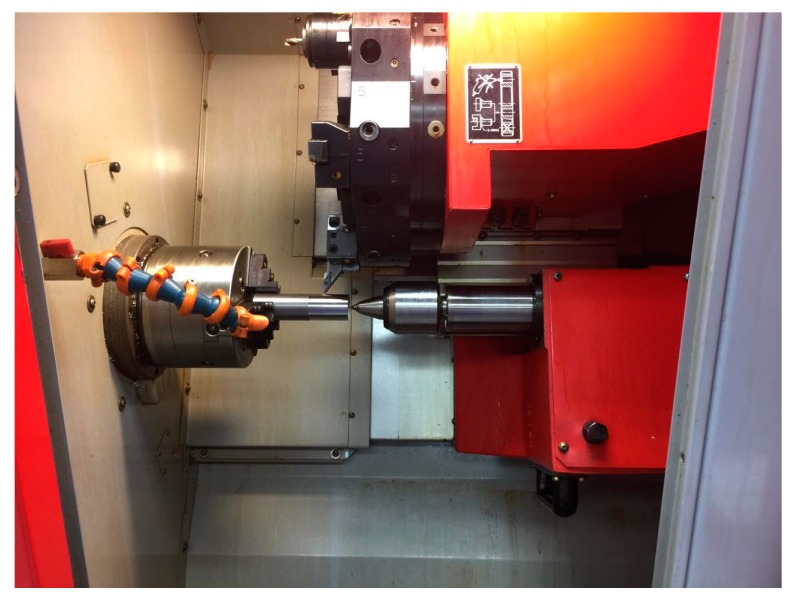
Test rig used for high-speed machining of Ti-6Al-4V.

The CNC Turning machine, Emco Concept Turn 45 (Austria), fitted with Sinumeric 840-D digital system (Siemens, Waltershausen, Germany) was used for machining. All machining tests were conducted through a CNC part program. The codes for the used tool holders and inserts (Sandvik, Sweden) are SVJCL2020K16 and VBMT160404-VBMT331-PM, respectively. The specifications of the inserts were nose radius: 0.4; clearance angle: 7°; and cutting-edge angle: 75°. All experiments were conducted with cutting fluid conditions using a cooling pump (2.2 KW). The test rig for machining the workpieces is shown in Figure 1. In this paper, the CNC machine power consumption was determined by connecting the machine’s power lines with two measuring types: Tactix 403057 multi-meters (Meridian International Co. Ltd., Hecho, China). The first one was used to measure the voltage (V), and the second one for measuring the current (I). The power (P) was calculated according to Equation (1).

(1) $$P=V\times I\times \sqrt{3}\cos\phi$$

where ϕ is the power factor, one multi-meter is connected to one power line to measure the current (I), and the other is connected to the other two power lines to measure the voltage (V). The load power factor was taken from the datasheet. The accuracy of the equipment was 2% at full scale. The power was averaged over three readings. Regarding measuring the surface roughness, a surface roughness apparatus was used to measure the surface roughness values (Rz). For measuring the tool flank wear (VB), a digital optical microscope equipped with digital readouts was used.

In terms of the design of the experiments, a full factorial array was used. Three speed levels (i.e., 100 m/min, 200 m/min, and 300 m/min), two levels of feed rate (i.e., 0.05 mm/rev and 0.15 mm/rev), two depth of cut levels (i.e., 0.1 mm and 0.3 mm), and four cutting lengths (i.e., 5 mm, 40 mm, 80 mm, and 120 mm) were included. Thus, a full factorial array (L48OA) was used to cover all possible combinations for reliable optimization purposes. In the next section, more details are provided for the multi-objective optimization methodology using the TOPSIS-fuzzy integrated approach.

## 3. Optimization Methodology

**Figure 2:**
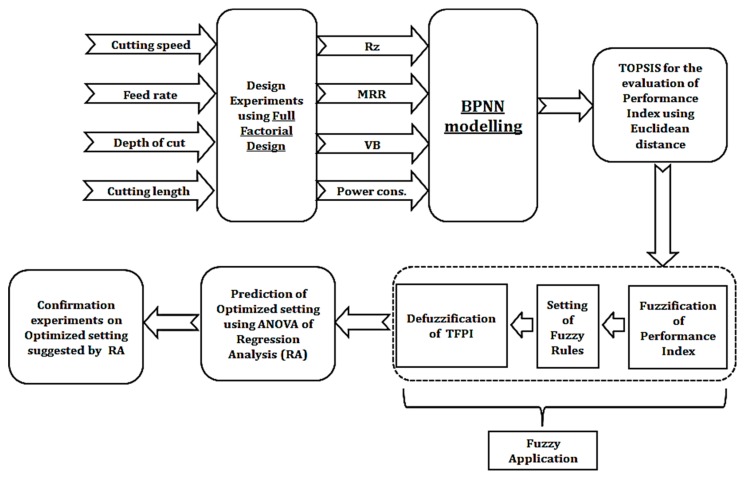
The overall methodology for modeling and optimization when high-speed machining Ti-6Al-4v.

This section describes the methodology used in the recent work for modeling and optimization of CNC machining parameters. Figure 2 presents the sequence of tasks conducted while machining Ti-6Al-4V.

**Figure 3:**
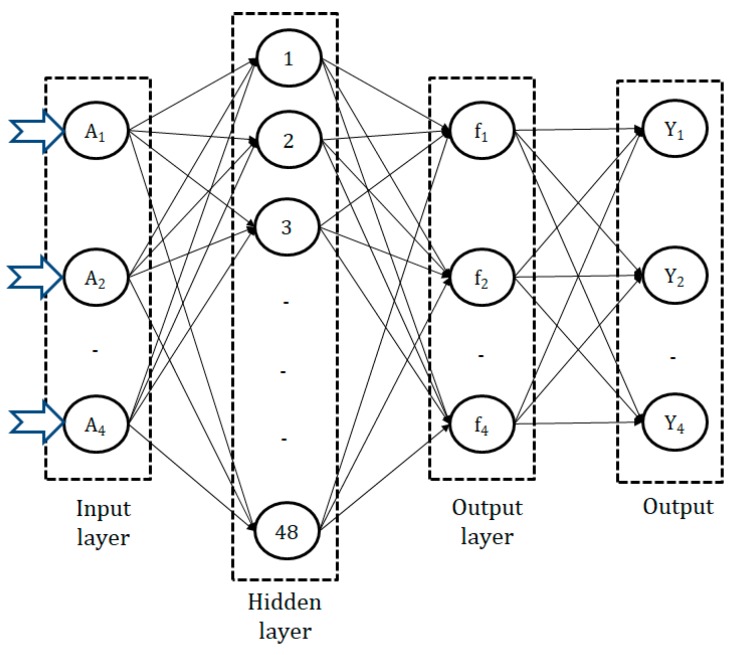
Architecture of the BPNN.

Backpropagation Neural Network (modeling step): A backpropagation concept with a complex system of weight space was first discussed by Kelley [14] and Bryson [15] for the minimization of errors. However, Dreyfus [16] used the simplified version of backpropagation coupled with a chain rule only. After that, the first neural network with a specific application of backpropagation was described by Werbos [17]. The publication of the related methodology started in 1985 with Parker [18]. In backpropagation neural networking, weights are assumed for the modeling purpose. The neural network is applied in such a way that it should resemble the very common element of biological neurons. Since it is affected by the biological system; therefore, the architecture of the neural network possesses a number of neurons and connections. The architecture of the backpropagation neural network (BPNN) consists of three layers, i.e., input layer, hidden layer, and output layer, which collectively provide the output with training, validation, and testing. Figure 3 depicts the architecture of BPNN according to the studied machining case model. In this architecture, cutting speed, feed rate, depth of cut, and cutting length are kept in the input layer while MRR, Rz, flank wear, and power consumption are in the output layer. 

Data Normalization using TOPSIS: The values of the response characteristics were varied in terms of magnitude and units. The normalization of data is a critical step in optimization problems. As the range of the response characteristics is very wide therefore normalization converts them into some comparable numbers. This helps to obtain a dimensionless number from the diverse measurement of response which is further utilized for ranking purposes in decision-making problems [19]. The below-mentioned steps were followed to convert the number of contrary responses into a single dimensionless performance index:

- In the first step, the attribute of the response variables is identified along with their categorization;
- In the second step, the normalized matrix is developed using Equation (2);
  (2) $${\bar{X}}_{\mathrm{ij}}=\frac{X_{\mathrm{ij}}}{\sqrt{\sum_{i=1}^{n}X_{\mathrm{ij}}^{2}}}$$
- In this step, the weighted normalized value is evaluated using Equation (3). The weights are predicted depending upon the importance of the respective response characteristics. In the present work, all the response characteristics were given equal importance, i.e., 0.33.
  (3) $$A_{\mathrm{ij}}={\bar{X}}_{\mathrm{ij}}\times W_{j}$$

Optimization with TOPSIS-Fuzzy: The fuzzy approach is basically utilized for uncertain data and can provide a solution for real-time applications. These applications have numerous advantages as they are easy to understand, allow quantitative comparison, and are less complex [20,21]. The implementation steps of hybrid the TOPSIS-fuzzy technique for computation of CNC machining parameters for optimum responses are as follows:

- In the first step, experimental data are initialized;
- In the second step, the normalization of the response characteristics are done using TOPSIS according to the weightage provided to each response;
- In the third step, the fuzzy logic system (FLS) is developed, where three sub-steps, namely, “fuzzification”, fuzzy rule development, and “defuzzification” are performed. In this step, the input data are converted into the degree of membership using one or more functions known as membership functions (MFs). Depending upon the categorization of the input and output data, the membership functions can be three, five, or even higher. Triangular MFs are always preferred due to the crisp value of the ultimate result. Therefore, the TOPSIS-fuzzy performance index (TFPI) is evaluated using the different MFs in the constant interval. Fuzzy rules are developed by the if-then control system based on “higher the better” type attributes [22]. These rules set a relationship between the inputs and output via a collection of philological statements [23]. The crisp value of TFPI is obtained after the incorporation of the center of gravity “defuzzification”.
- The crisp values of the TFPI obtained in the third step were analyzed by the statistical solver tool Minitab using analysis of variance (ANOVA), and empirical models were developed using regression analysis.

## 4. Results and Discussions

Table 3 presents the experimental matrix (i.e., experimental combinations of process parameters) and corresponding values of responses. This was used as a source of data for the TOPSIS normalization.

### 4.1. BPNN Modeling

The main mechanism running behind backpropagation is the partial derivative ∂C/∂w. Here, “C” is the cost function, and “w” is any weight (or bias “b”) in the network. This partial derivative defines how quickly the cost of any function varies after a small variation in the weight (or bias). The significant advantage of BPNN is that each element has a spontaneous and natural interpretation if complexity exists. Therefore, BPNN not only provides a fast processing algorithm but also provides insights. The calculations in this take place as per Equations (4)–(6).

(4) $$Y_{\mathrm{New}}=\frac{D_{i}-\mu }{k\times \sigma }\;$$

where µ is the data’s mean, σ is the standard deviation of the data, and k = 2.58 (for 99%).

(5) $$Y_{\mathrm{New}}=\left[\frac{D_{i}-\mathrm{Min}}{\mathrm{Max}-\mathrm{Min}}\;\left(0.8-0.2\right)+0.2\right]$$

(6) $$\sigma =\sigma_{\min}+\left(p-1\right)\frac{\sigma_{\max}-\sigma_{\min}}{N}$$

where, p = 1, 2, 3… 48; σ_min_ = 1; σ_max_ = 48; N = 48.

The following steps were taken for the BPNN modeling. In the first step, 48 experimental data sets were specified. Sixty percent of the data from those specified data sets were used for training, 20% for validation, and 20% for checking the planned model; In the next step, the smoothing parameter was acquired by a technique called cross-validation. The optimum adaptive parameter (σ) was assessed to the keep cross-validation error to a minimum using a grid search technique. In the third step, the scalar function (Yi) was obtained, where i = 1 to 48 and shows the entire experimental data sets. In the fourth step, the coefficient was calculated using Equation (4), incorporating the values of Di and σ. In the radial layer through step 5, the real output data set Y_i_ was multiplied with the exponential term, respectively. In the sixth step, the probability density function (PDF) was calculated with the help of spare neurons in the regression layer. The successively weighted average of the BPNN output parameters was calculated within the experimental array. This last step was validation and testing, as mentioned in Step 1, and 40% of the data was used for this purpose. The kernel width (σ) was the only adaptive parameter, where a smaller value showed overfitting of the data, and a higher value showed over smoothing. So, the optimum value of kernel width was obtained using the grid search method. Equation (5) was used for the execution of cross-validation of kernel width for obtained “N” values.

**Figure 4:**
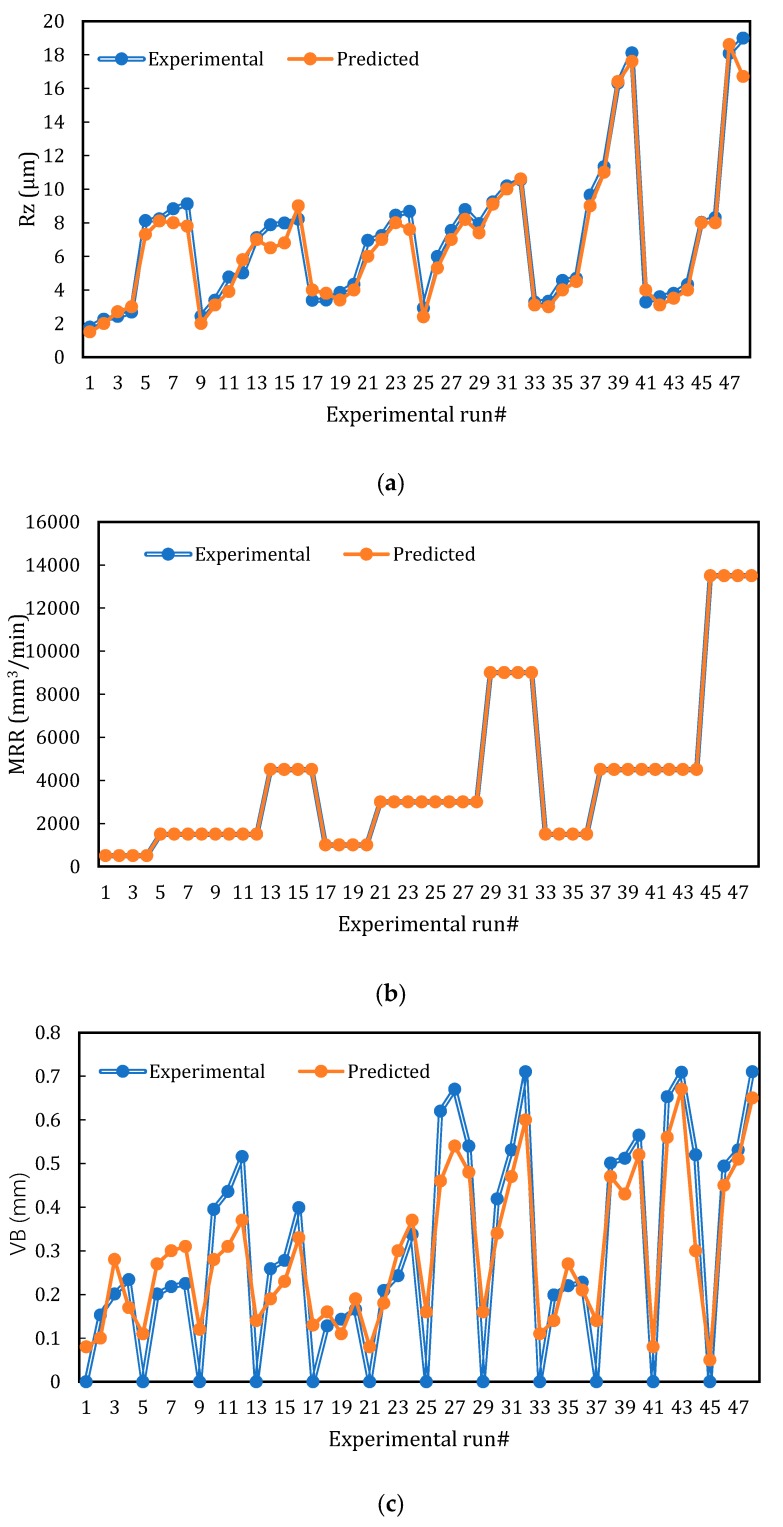

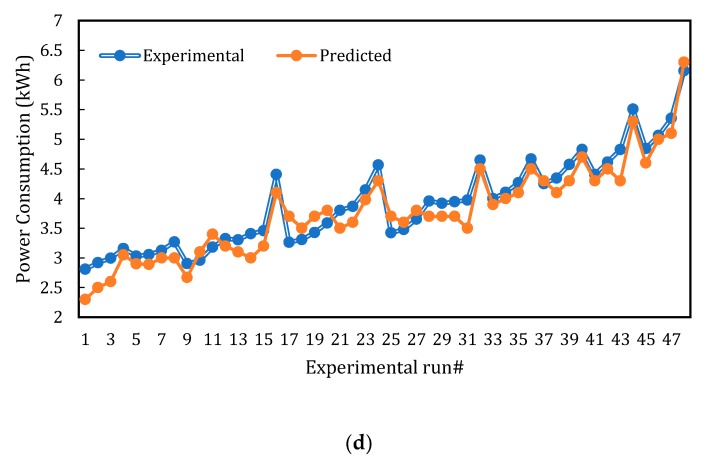
Predicted versus experimental runs: (**a**) Rz; (**b**) MRR; (**c**) VB; (**d**) power consumption (modeling step using BPNN).

Figure 4 presents the correlation between the experimental values of Rz, MRR, VB, power consumption and the predicted values by BPNN. A close agreement between the experimental and predicted values was observed which proves the effectiveness of the BPNN modeling to establish the relationship between the studied machining parameters and responses and their further prediction with minimum error for ready industrial use.

### 4.2. Multi-Objective Optimization Results

After experimentation, the optimal machining parameters for contradictory response characteristics (i.e., MRR, the higher the better; Rz; VB; power, the lower the better) have been investigated by a TOPSIS-fuzzy-based hybrid approach. The implementation procedure has already been discussed in the optimization methodology section. The decision matrix was initialized, and the normalization was processed according to Equations (1) and (2), providing equal weight to each response. Once the normalized values of response characteristics were obtained using TOPSIS, the fuzzy model was developed using the fuzzy interface system (FIS). “Mamdani”-type FIS provides a crisp value of the performance index (PI) by developing a membership function (MF) for input and output parameters. In the present research, a three-level fuzzy sub-set (i.e., small, medium, and large) of equal intervals was assumed for MRR, VB, and power consumption. However, for Rz, five levels were set (i.e., very small, small, medium, large, and very large). Whereas, the output response, namely, TOPSIS-fuzzy performance index (TFPI), was investigated by a seven-level subset (i.e., extremely small, very small, small, medium, large, very large, and extremely large) of equal intervals. The fuzzy rules were established to determine “TFPI” using the “if–then” statement. As per the number of levels assumed in MF, 45 rules were formed to develop the fuzzy model by the knowledge of control engineering. An example of the fuzzy rules used in the present work is described as follows:

“Rule 1: If Rz is very small and MRR is small, and VB is small, and power consumption is small, then TFPI is extremely small”.

The performance index after the “defuzzification” of supplied normalized input and output was generated in the form of TFPI. The values of TFPI, as predicted by the hybrid TOPSIS-fuzzy technique, are shown in Table 4. The first four columns represent the input of weighted normalized values corresponding to Rz, MRR, and VB, while the last column corresponds to the predicted TFPI.

The TFPI value obtained by the TOPSIS-fuzzy method was further processed by regression analysis to convert it into an empirical relation. The regression analysis of TFPI gives the regression coefficients for input parameters, and the corresponding empirical model is provided in Equation (7).

TFPI = 0.1586 – 0.000118v – 0.1135d – 0.026f + 0.00016l(7)

**Figure 5:**
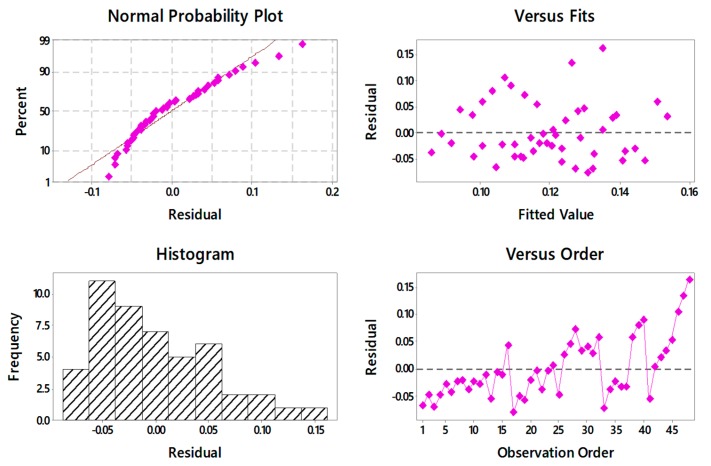
Plots of the residuals for TFPI.

Figure 5 shows the residual plots for the developed empirical model. All the residuals were found on a straight line which reveals the normal distribution of residual and verifies the normality test. In the fit versus residual plot, all the residuals should be randomly distributed for an appropriate ANOVA. In the present research, this test was found to be a good fit with the experimental results. The best ANOVA is usually defined by the parabolic histogram which was observed in the present work. The fourth ANOVA test of time–variance was also satisfied in the current research. The larger the TFPI, the better the quality of the attribute; therefore, the highest level of process parameters suggests the better TFPI value. It was found that the third level of cutting speed (i.e., 300 m/min), the second level of depth of cut (i.e., 0.3 mm), the second level of feed rate (i.e., 0.15 mm/rev), and the second level of cutting length (i.e., 40 mm) provided the maximum TFPI. Therefore, the response characteristics corresponding to this setting provided the optimal solution which achieved the highest balance among all studied outputs.

### 4.3. Results Validation

The investigated optimal solution obtained from the integrated approach was verified with an experimental test to validate the effectiveness of the used optimization approach. The proposed methodology with TFPI predicted that a cutting speed of 300 m/min, depth of cut of 0.3 mm, feed rate of 0.15 mm/rev, and a cutting length 40 mm were the optimal machining parameters setting. A comparison between the predicted and experimental values at the abovementioned cutting conditions is provided in Table 5. It should be stated that the predicted values for all machining outputs demonstrated an excellent agreement with the experimental values at the selected optimal solution (run 46: v = 300 m/min, f = 0.15 mm/rev, d = 0.15 mm, and l = 40 mm). It also should be stated that the selected optimal solution did not provide the best performance for each measured output, as can be seen in Table 5, but it achieved a balance among all studied responses. It can be seen that the selected solution achieved the highest possible MRR; however, it provided somehow higher values for both VB as well as Rz while consuming a medium level of power. In this study, equally weighted values were considered. However, if the required solution needed to achieve a certain level of surface quality or power consumption with less concern about the productivity aspects, customized weights for the studied machining outputs should be considered to provide different optimal solutions. In addition, based on the results provided in Table 4 and Table 5, any other solution can be selected according to the decision-maker’s criteria, for example, run 41 offered a promising performance in terms of tool wear and surface quality compared to other tests (i.e., Rz = 3.281 µm and VB = 0.2 mm); however, the productivity was lower than the optimal solution provided in Table 5, and accordingly its TFPI was less than the optimal solution. Thus, it should be stated that the applied optimized method was not only able to provide an estimate regarding the optimal solution (highest TFPI), but it also offered a mapping system for other solutions which can be beneficial to the decision making selecting the appropriate cutting conditions according to the desired criteria. To sum up, the integrated approach showed effective capabilities to provide the optimal solution (optimal cutting conditions) which achieved a reasonable balance among all studied responses when machining Ti-6Al-4V. This is an important step towards a reliable machining system as obtained in the open literature [24,25,26].

## 5. Conclusions and Future Work

In this work, an experimental investigation into the influence of different machining parameters (i.e., cutting speed, feed rate, depth of cut, and cutting length) on surface roughness (Rz), flank wear (VB), power consumption as well as material removal rate (MRR) when high speed turning Ti-6Al-4V alloy was presented and discussed. In addition, BPNN along with the TOPSIS-fuzzy integrated approach was employed to model and optimize the overall cutting performance. A close agreement between the experimental and predicted values was observed which proves the effectiveness of the BPNN modeling to establish the relationship among the studied machining parameters and responses and their further prediction with minimum error for ready industrial use. In terms of the optimization results, it was found that the third level of cutting speed of 300m/min, the second level of depth of cut at 0.3 mm, the second level of feed rate of 0.15 mm/rev, and the second level of cutting length of 40 mm provided the maximum TFPI. Therefore, the response characteristics corresponding to this setting provided the optimal solutions which achieved the highest balance among all studied outputs. It should be stated that the predicted values for all machining outputs demonstrated an excellent agreement with the experimental values at the selected optimal solution (v = 300 m/min, f = 0.15 mm/rev, d = 0.15 mm, and l = 40 mm). It also should be stated that the selected optimal solution did not provide the best performance for each measured output, but it achieved a balance among all studied responses. To sum up, the applied optimized approach was not only able to provide an estimate about the optimal solution (highest TFPI), but it also offered a mapping system for other solutions which can simplify the selection of the appropriate cutting conditions according to the desired criteria. Regarding future work, the sustainability effect in terms of machining cost, environmental impact, and waste management will be included and optimized.

## Figures and Tables

**Table 1:** Chemical composition of titanium-6aluminum-4vanadium (Ti-6Al-4V).

N	C	H	Fe	O	Al	V	Ti
0.05	0.1	0.012	0.4	0.2	6	4	Balance

**Table 2:** Mechanical properties of Ti-6Al-4V.

Tensile Strength, (MPa)	Yield Strength, 0.2% Offset, (MPa)	Elongation, (%)	Reduction of Area, (%)	Hardness
895	825	10	25	HRC36

**Table 3:** Experimental results for the machining of Ti-6Al-4V.

#	v (m/min)	d (mm)	f (mm/rev)	l (mm)	Rz (µm)	MRR (mm^3^/min)	VB (mm)	Power Consumption (kWh)
1	100	0.1	0.05	5	1.786	500	0.01	2.808
2	100	0.1	0.05	40	2.257	500	0.153	2.919
3	100	0.1	0.05	80	2.421	500	0.201	2.995
4	100	0.1	0.05	120	2.681	500	0.234	3.157
5	100	0.1	0.15	5	8.124	1500	0.02	3.031
6	100	0.1	0.15	40	8.232	1500	0.201	3.054
7	100	0.1	0.15	80	8.829	1500	0.218	3.126
8	100	0.1	0.15	120	9.12	1500	0.225	3.269
9	100	0.3	0.05	5	2.432	1500	0.01	2.903
10	100	0.3	0.05	40	3.384	1500	0.395	2.959
11	100	0.3	0.05	80	4.762	1500	0.436	3.182
12	100	0.3	0.05	120	5.003	1500	0.516	3.324
13	100	0.3	0.15	5	7.107	4500	0.03	3.305
14	100	0.3	0.15	40	7.876	4500	0.259	3.407
15	100	0.3	0.15	80	7.983	4500	0.278	3.458
16	100	0.3	0.15	120	8.211	4500	0.399	4.409
17	200	0.1	0.05	5	3.386	1000	0.01	3.263
18	200	0.1	0.05	40	3.402	1000	0.128	3.309
19	200	0.1	0.05	80	3.846	1000	0.143	3.427
20	200	0.1	0.05	120	4.337	1000	0.166	3.587
21	200	0.1	0.15	5	6.949	3000	0.02	3.802
22	200	0.1	0.15	40	7.233	3000	0.209	3.868
23	200	0.1	0.15	80	8.446	3000	0.243	4.145
24	200	0.1	0.15	120	8.677	3000	0.338	4.568
25	200	0.3	0.05	5	2.92	3000	0.01	3.424
26	200	0.3	0.05	40	5.987	3000	0.62	3.478
27	200	0.3	0.05	80	7.538	3000	0.67	3.654
28	200	0.3	0.05	120	8.78	3000	0.54	3.958
29	200	0.3	0.15	5	7.938	9000	0.03	3.921
30	200	0.3	0.15	40	9.236	9000	0.419	3.946
31	200	0.3	0.15	80	10.191	9000	0.531	3.975
32	200	0.3	0.15	120	10.543	9000	0.71	4.649
33	300	0.1	0.05	5	3.273	1500	0.02	4
34	300	0.1	0.05	40	3.323	1500	0.199	4.105
35	300	0.1	0.05	80	4.567	1500	0.22	4.267
36	300	0.1	0.05	120	4.684	1500	0.228	4.669
37	300	0.1	0.15	5	9.642	4500	0.03	4.252
38	300	0.1	0.15	40	11.327	4500	0.501	4.345
39	300	0.1	0.15	80	16.301	4500	0.512	4.575
40	300	0.1	0.15	120	18.101	4500	0.565	4.829
41	300	0.3	0.05	5	3.281	4500	0.02	4.406
42	300	0.3	0.05	40	3.592	4500	0.653	4.615
43	300	0.3	0.05	80	3.788	4500	0.709	4.826
44	300	0.3	0.05	120	4.326	4500	0.52	5.508
45	300	0.3	0.15	5	8.018	13500	0.01	4.844
46	300	0.3	0.15	40	8.3	13500	0.494	5.065
47	300	0.3	0.15	80	18.07	13500	0.531	5.354
48	300	0.3	0.15	120	18.98	13500	0.71	6.156

**Table 4:** TOPSIS normalized results and fuzzy-based performance indices.

#	Weighted Normalized				TFPI
	Rz	MRR	VB	Power Consumption	
1	0.0079	0.0033	0.0000	0.0254	0.0366
2	0.0099	0.0033	0.0118	0.0264	0.0515
3	0.0107	0.0033	0.0156	0.0271	0.0566
4	0.0118	0.0033	0.0181	0.0285	0.0618
5	0.0358	0.0100	0.0000	0.0274	0.0732
6	0.0362	0.0100	0.0156	0.0276	0.0894
7	0.0389	0.0100	0.0169	0.0282	0.0940
8	0.0401	0.0100	0.0174	0.0295	0.0971
9	0.0107	0.0100	0.0000	0.0262	0.0470
10	0.0149	0.0100	0.0306	0.0267	0.0822
11	0.0210	0.0100	0.0338	0.0288	0.0935
12	0.0220	0.0100	0.0399	0.0300	0.1020
13	0.0313	0.0301	0.0000	0.0299	0.0912
14	0.0347	0.0301	0.0200	0.0308	0.1156
15	0.0351	0.0301	0.0215	0.0312	0.1180
16	0.0361	0.0301	0.0309	0.0398	0.1369
17	0.0149	0.0067	0.0000	0.0295	0.0511
18	0.0150	0.0067	0.0099	0.0299	0.0615
19	0.0169	0.0067	0.0111	0.0310	0.0656
20	0.0191	0.0067	0.0128	0.0324	0.0710
21	0.0306	0.0200	0.0000	0.0344	0.0850
22	0.0318	0.0200	0.0162	0.0349	0.1030
23	0.0372	0.0200	0.0188	0.0375	0.1135
24	0.0382	0.0200	0.0262	0.0413	0.1257
25	0.0129	0.0200	0.0000	0.0309	0.0638
26	0.0264	0.0200	0.0863	0.0314	0.1641
27	0.0332	0.0200	0.0882	0.0330	0.1745
28	0.0386	0.0200	0.0889	0.0358	0.1834
29	0.0349	0.0601	0.0000	0.0354	0.1305
30	0.0407	0.0601	0.0324	0.0357	0.1689
31	0.0449	0.0601	0.0411	0.0359	0.1820
32	0.0464	0.0601	0.0599	0.0420	0.2085
33	0.0144	0.0100	0.0000	0.0361	0.0606
34	0.0146	0.0100	0.0154	0.0371	0.0771
35	0.0201	0.0100	0.0170	0.0386	0.0857
36	0.0206	0.0100	0.0176	0.0422	0.0905
37	0.0424	0.0301	0.0000	0.0384	0.1109
38	0.0499	0.0301	0.0388	0.0393	0.1580
39	0.0717	0.0301	0.0396	0.0413	0.1828
40	0.0797	0.0301	0.0437	0.0436	0.1971
41	0.0144	0.0301	0.0000	0.0398	0.0843
42	0.0158	0.0301	0.0505	0.0417	0.1381
43	0.0167	0.0301	0.0549	0.0436	0.1452
44	0.0190	0.0301	0.0721	0.0498	0.1710
45	0.0353	0.0902	0.0000	0.0438	0.1693
46	0.0665	0.1202	0.0582	0.0658	0.3107
47	0.0795	0.0902	0.0411	0.0484	0.2592
48	0.0835	0.0902	0.0675	0.0556	0.2969

**Table 5:** Results validation and comparison with the optimal experimental values.

	Experimental Values	Predicted TOPSIS-Fuzzy	Optimal Experimental Value
	v = 300 m/min, f = 0.15 mm/rev, d = 0.15 mm, and l = 40 mm		
Rz (µm)	8.311	8.032	1.786
MRR (mm^3^/min)	13500	13500	13500
VB (mm)	0.494	0.45	0.01
Power Consumption (kWh)	5.065	5.21	2.808
